# Clinical characteristics and risk factors for developing bone metastases in patients with breast cancer

**DOI:** 10.1038/s41598-017-11700-4

**Published:** 2017-09-12

**Authors:** Wen-Zhao Chen, Jun-Feng Shen, Yang Zhou, Xuan-Yin Chen, Jia-Ming Liu, Zhi-Li Liu

**Affiliations:** Department of Orthopaedic Surgery, the First Affiliated Hospital of Nanchang University, Nanchang, 330006 PR China

## Abstract

The risk factors for predicting bone metastases in patients with breast cancer are still controversial. Here, a total of 2133 patients with breast cancer, including 327 with bone metastases (15.33%) and 1806 without bone metastases (84.67%) were retrospective reviewed from January 2005 to December 2015. The spine was found to be the most common site for bone metastases, followed by ribs (57.5%), pelvis (54.1%) and sternum (44.3%). The results indicated that axillary lymph node metastases and the concentrations of CA125, CA153, ALP and hemoglobin were the independent risk factors for bone metastases in patients with breast cancer. The receiver operating characteristics (ROC) curves showed that combined axillary lymph node metastases, high CA153 and ALP, with low hemoglobin were the most accurate biomarkers for predicting bone metastases in breast cancer [area under the curve = 0.900], and the sensitivity and specificity for the prediction were 78.5% and 87.8%, respectively. Therefore, breast cancer patients with more axillary lymph node metastases, high serum concentrations of CA125, CA153, ALP and low level of hemoglobin were closely related to bone metastases. Combined axillary lymph node metastases, CA153, ALP with hemoglobin have the highest predictive accuracy for bone metastases in breast cancer.

## Introduction

Breast cancer is the most common malignant cancer in women and is the leading cause of cancer death for women worldwide. Due to the early detection by mammography, better understanding of the natural history and the improvement of surgical and medical therapies for breast cancer, the prognosis and survival rate of it has improved during the last decade. However, women with breast cancer are vulnerable to develop metastatic diseases during the early stage^1, 2^.

Bone is the most frequent site of distant metastasis from breast cancer and accounts for the highest proportion of first site relapse in patients with breast cancer. The rate of five-year survival will be significantly decreased and severe complications will present following bone metastases in breast cancer patients^3^. In addition, bone metastases are predominant osteolytic in breast cancer. Osteolytic metastases can cause dramatic bone loss, which finally resulted in skeletal related events (SREs), such as pathological fractures, severe pain, bone instability, spinal cord compression and hypercalcemia. These will reduce the quality of patients’ life^3^. Therefore, early detection and diagnosis of bone metastasis in patients with breast cancer is helpful for the treatment.

Bone scintigraphy (BS) is a traditionally sensitive and efficient method for initial evaluation and follow-up of bone metastases, although it is considered to be a nonspecific method. However, the high expenditure and the radiation for patients due to repeat using BS for continuous evaluation of bone metastases can’t be ignored^4^. Thus, improving knowledge about the clinical characteristics and identification of the risk factors for bone metastases in breast cancer patients is important for early diagnosing and treatment.

In this retrospective study, we tried to evaluate the incidence and distribution of bone metastases in breast cancer. Also, we aimed to investigate the correlation between clinical-pathological parameters and bone metastases, and identify the risk factors for bone metastases in patients with breast cancer.

## Materials and Methods

### Study design

This study was approved by the Ethics Committee of the First Affiliated Hospital of Nanchang University, and all participants provided written informed consent. All methods were performed in accordance with relevant guidelines and regulations. A retrospective study was carried out and patients who were diagnosed as breast cancer at our hospital between January 2005 and December 2015 were included in this study. The diagnosis was made based on the histopathological analysis of patients specimens harvested by biopsy or surgical resection. Bone metastasis and other organs metastases were identified by bone scanning. If necessary, local computed tomography (CT) and MRI would be performed to confirm the diagnosis. Patients with bone metabolic diseases, renal failure, feeding disorders and a second primary malignancy were excluded from this study. In addition, breast patients with other distant organs metastases, such as lung, liver and brain metastases, were also excluded from this study.

### Data collection

All patients’ clinical data were obtained from the medical record, such as age, gender, menopausal status, histopathological subtype, axillary nodal status, serum concentrations of calcium, alkaline phosphatase (ALP), carcinoembryonic antigen (CEA), cancer antigen 125 (CA 125), cancer antigen 153 (CA153) and cancer antigen 199 (CA199) at the time of primary diagnosis. The incidence and distribution of bone metastases from breast cancer were evaluated, and the correlation between diverse clinical-pathological parameters and bone metastases were analyzed in the study.

### Statistical analysis

All the statistical analysis was performed by SPSS19.0 software (Chicago, USA). Continuous data were demonstrated as means ± standard deviation. First, the Chi-square test and student t test were used to detect the differences between patients with and without bone metastases. Binary logistic regression model was then established to identify the independent risk factors for bone metastases in breast cancer. Receiver operating characteristics (ROC) curves were plotted and areas under the curve (AUC) were calculated to evaluate the predictive accuracy of risk factors for predicting bone metastases. The ROC analysis was firstly conducted for individual factor and then for the combination factors. A *P* < 0.05 was considered to be statistically significant.

## Results

### Patient’s clinical characteristics

A total of 2133 patients were recruited in this study, including 327 with bone metastases (15.33%) and 1806 without bone metastases (84.67%) at the primary diagnosis. Of these patients, 46 were firstly diagnosed with bone metastases and then were identified with primary breast cancer. Patients’ clinical characteristics were illustrated in Table 1. Most of breast cancer patients were at the age of 40 to 49 years. And the mean age of patients with and without bone metastases were 47.29 ± 10.59 and 48.13 ± 10.60 years (*P* = 0.185), respectively. Histopathological subtype was predominated by invasive ductal carcinoma (80.73%) in these patients. At the time of diagnosis, 63.89% of the patients were at the status of premenopausal and 63.31% of them had one or more positive axillary lymph nodes metastases.

**Table 1:** The clinical characteristics of patients with breast cancer.

Characteristics	Total number of patients (%)	Bone metastases group (n = 327)	Non metastases group (n = 1806)	P* value
**Age (years)**	48.01 ± 10.60	47.29 ± 10.59	48.13 ± 10.60	0.185
<30	54 (2.5)	11	43	0.828
30–34	118 (5.5)	19	99	
35–39	271 (12.7)	49	222	
40–44	409 (19.2)	59	350	
45–49	415 (19.5)	63	352	
50–54	349 (16.4)	53	296	
55–59	217 (10.2)	30	187	
>59	300 (14.1)	43	257	
**Gender (n)**				0.278
Woman	2116 (99.2)	326	1790	
Man	17 (0.8)	1	16	
**Menopausal status (n)**				0.275
Premenopausal	1352 (63.9)	217	1135	
Postmenopausal	764 (36.1)	109	655	
**Histopathology (n)**				0.098
Invasive ductal carcinoma	1722 (80.7)	273	1449	
Other types	286 (13.4)	32	254	
Unknown	125 (5.9)			
**Axillary lymph node metastases# (n)**				<0.001
N0	485 (36.69)	35	450	
N1–3	481 (36.38)	112	369	
N4 and more	356 (26.93)	130	226	

*P value: comparison between bone metastases group and non bone metastases group. ^#^N0: without axillary lymph node metastasis; N1–3: 1 to 3 lymph nodes metastases; N4 and more: 4 and more than 4 lymph nodes metastases.

### Distribution of bone metastases in patients with breast cancer

For the distribution of bone metastases in breast cancer, the most frequent site was spine, including thoracic spine (63.61%) and lumbar spine (53.82%). The second commonly metastatic site was ribs (57.49%), and then followed by pelvis (54.1%) and sternum (44.34%). The least affected site of bone metastases were ulna and radius (0.31%) (Table 2).

**Table 2:** The distribution of bone metastases in patients with breast cancer.

Metastases sites	Number of patients (n = 327)
Thoracic spine	208 (63.6%)
Lumbar spine	176 (53.8%)
Cervical spine	71 (21.7%)
Rib	188 (57.5%)
Pelvis	177 (54.1%)
Sternum	145 (44.3%)
Scapula	82 (25.1%)
Femur	81 (24.8%)
Humerus	70 (21.41%)
Skull	68 (20.8%)
Clavicle	43 (13.2%)
Tibia and fibula	9 (2.8%)
Ulna and radius	1 (0.3%)

### Risk factors for bone metastases in breast cancer

In order to identify the potential risk factors for bone metastases, Chi-square test for dichotomous data and student t test for continuous data were used for the analysis. The results showed that significant difference was found on axillary lymph node metastases between patients with and without bone metastases (*P* < 0.001). And the serum concentrations of CEA, CA125, CA153, CA199 and ALP were significantly higher in patients with bone metastases than those without bone metastases (*P* < 0.05, respectively). However, the serum level of hemoglobin in bone metastatic patients was statistically lower than those non bone metastases (*P* < 0.001) (Tables 1 and 3). Additionally, binary logistic regression analysis indicated that axillary lymph node metastases, CA125, CA153, ALP and hemoglobin were the independent risk factors for bone metastases in patients with breast cancer (*P* < 0.05, respectively) (Table 4).

**Table 3:** The differences of clinical parameters between patients with and without bone metastases.

Clinical factors	Bone metastases group	Non metastases group	*P* value
CEA (ng/ml)	56.68 ± 157.25	3.97 ± 17.23	<0.001
CA125 (u/ml)	120.44 ± 266.67	32.56 ± 147.29	<0.001
CA153 (u/ml)	174.05 ± 352.13	19.18 ± 38.11	<0.001
CA199 (u/ml)	168.09 ± 792.90	20.58 ± 113.65	0.007
ALP (u/l)	170.33 ± 136.22	64.69 ± 28.34	<0.001
Calcium (mmol/l)	2.31 ± 0.24	2.31 ± 0.16	0.763
Hb (g/l)	108.50 ± 17.32	118.67 ± 14.25	<0.001

**Table 4:** The risk factors for predicting bone metastases in patients with breast cancer.

Factors	B	OR	OR (95% CI)	P
Axillary lymph node metastases	−0.319	0.727	0.581–0.910	0.005
CA125	0.001	1.001	1.000–1.003	0.041
CA153	−0.019	0.981	0.974–0.989	<0.001
ALP	−0.025	0.976	0.970–0.981	<0.001
HB	0.021	1.021	1.008–1.034	0.002

Note: B: coefficient of regression, OR: odds ratio, CI: confidence interval.

### The cut-off values, sensitivities and specificities of risk factors for diagnosing bone metastases

**Figure 1:**
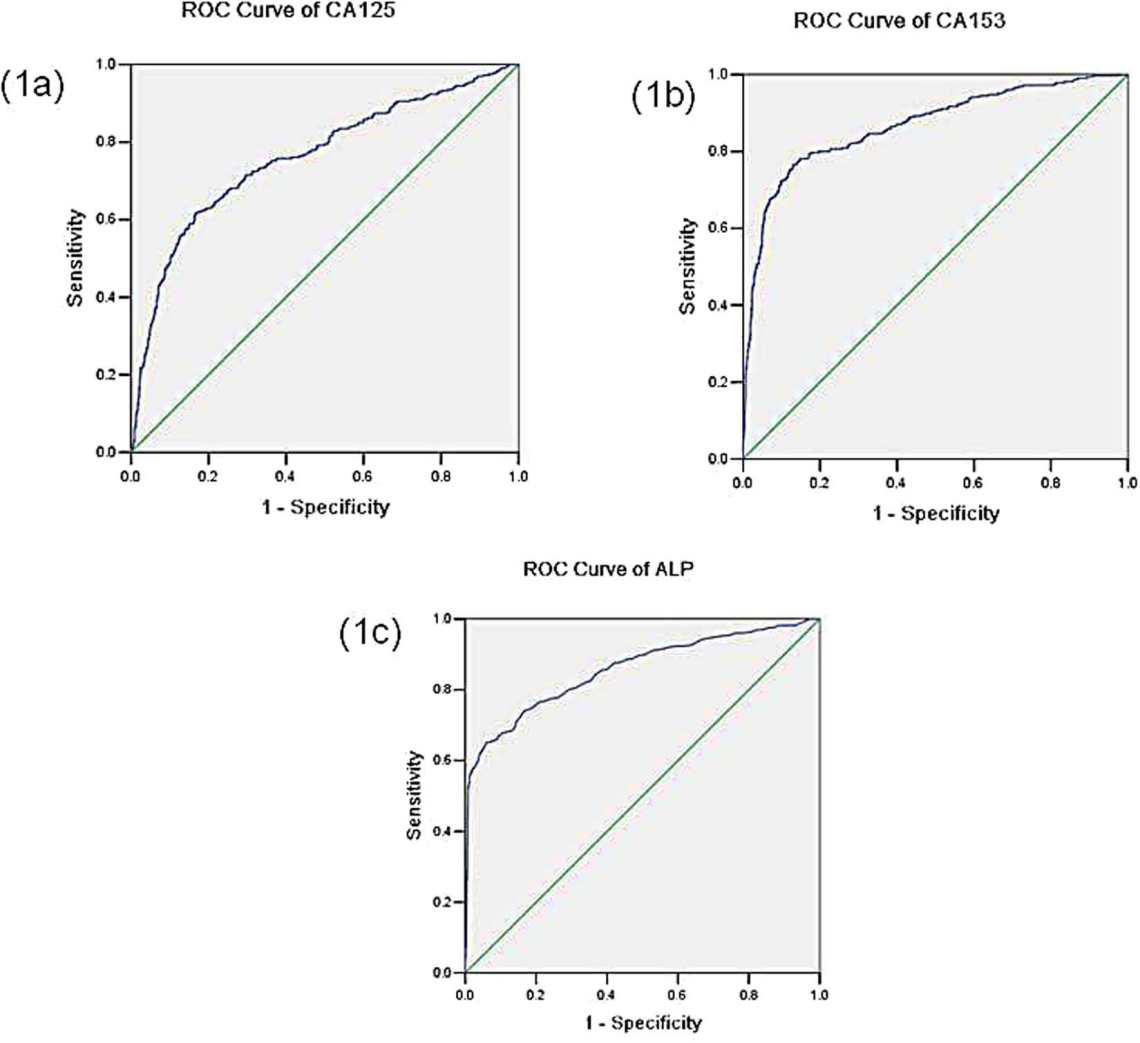
The receiver operating characteristics (ROC) curves of risk factors for detecting bone metastases in breast cancer. (**a**), the ROC curve of CA125. (**b**) the ROC curve of CA153. (**c**) ROC curve of ALP.

The ROC curve was established to determine the accuracy, sensitivities and specificities of predicting bone metastases by these risk factors (Fig. 1 and Table 5). Based on the analysis, the concentration of CA153 had the highest predictive accuracy for bone metastases among these factors (AUC = 0.866), with a sensitivity of 76.62% and specificity of 86.97%. However, as a single risk factor, hemoglobin had a low accuracy on predicting bone metastases (AUC = 0.316). The cut-off values of CA125, CA153, ALP and hemoglobin for the prediction were 21.99 u/ml, 25.42 u/ml, 100.5 u/l and 49 g/l, respectively. Thus, patients with CA125 >21.99 u/ml, CA153 >25.42 u/ml, ALP >100.5 u/l and hemoglobin <49 g/l were more closely correlated to bone metastases.

**Table 5:** The cutoff value, sensitivity and specificity of single risk factor for predicting bone metastasis in breast cancer.

Factors	Cutoff value	Sensitivity (%)	Specificity (%)	AUC	*P*
CA125 (u/ml)	21.99	61.66	83.36	0.761	<0.001
CA153 (u/ml)	25.42	76.62	86.97	0.866	<0.001
ALP (u/l)	100.5	65.14	93.96	0.856	<0.001
HB (g/l)	49	100	0.23	0.316	<0.001

Note: AUC, area under the curve; HB, hemoglobin.

### Combination of different factors for diagnosing bone metastases in breast cancer

**Figure 2:**
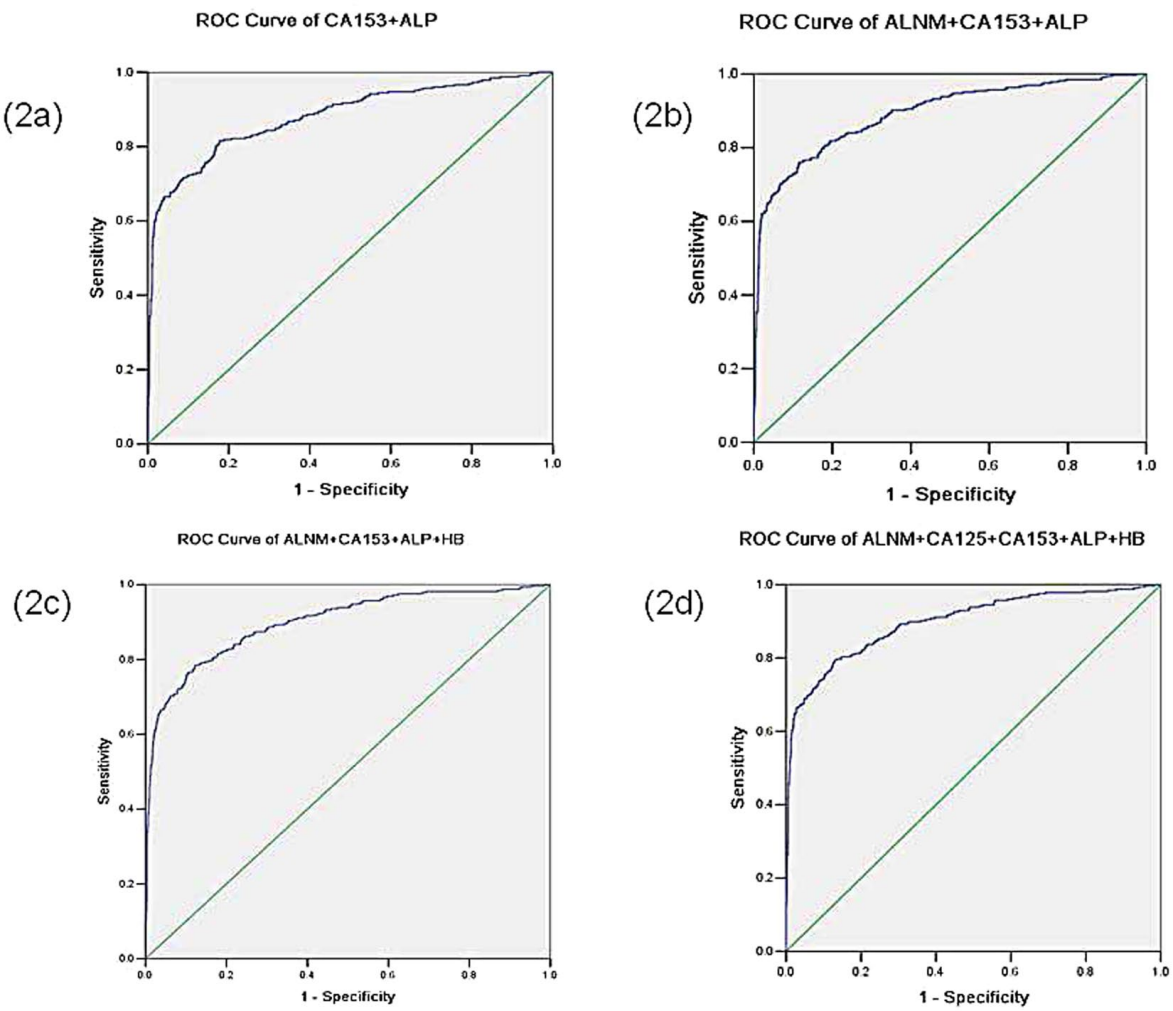
The receiver operating characteristics (ROC) of different combinations of risk factors for diagnosing bone metastases. (**a**) the ROC curve of CA153 + ALP. (**b**) the ROC curve of axially lymph node metastases + CA153 + ALP. (**c**) the ROC curve of axially lymph node metastases + CA153 + ALP + hemoglobin. (**d**) the ROC curve of axially lymph node metastases + CA125 + CA153 + ALP + hemoglobin.

In order to investigate the predictive accuracy of combining risk factors for bone metastases in patients with breast cancer, ROC analysis was conducted for different combination of risk factors (Table 6 and Fig. 2). For two combinations of risk factors, combined CA153 with ALP were more accurate than other combinations for the prediction (AUC = 0.881). Among the three combinations of risk factors, combined axially lymph node metastases, CA153 and ALP were the most accurate one for prediciting bone metastases (AUC = 0.892). Additionally, combined axially lymph node metastases, CA153, ALP and hemoglobin had the highest predictive value among the four combinations of risk factors (AUC = 0.900). Comparison of all the combining risk factors for the diagnosing, four combinations of axially lymph node metastases, CA153, ALP and hemoglobin were the most accurate one for predicting bone metastases in breast cancer, with a sensitivity of 78.5% and specificity of 87.8%.

**Table 6:** The analysis of predictive accuracy of combining different risk factors for bone metastases in breast cancer.

Combined factors	AUC	Sensitivity (%)	Specificity (%)	*P*
CA153 + ALP	0.881	81.5	82.3	<0.001
ALNM + CA153 + ALP	0.892	76.0	88.4	<0.001
ALNM + CA153 + ALP + HB	0.900	78.5	87.8	<0.001
ALNM + CA125 + CA153 + ALP + HB	0.899	79.4	87.0	<0.001

Note: AUC, area under the curve; ALNM: Axillary lymph node metastases; HB, hemoglobin.

## Discussion

Bone is one of the most common sites of distant metastases in breast cancer and usually associated with a poor prognosis and low quality of life for patients. The CT, positron emission tomography–computed tomography (PET-CT) and MRI are time-consuming and costly for patients, limited by low sensitivity and specificity. Bone scan is a popular method for detecting distant bone metastases in patients with tumor, but it is unable to identify osteolytic metastasis and is usually affected by the flare phenomenon^5, 6^. Therefore, early diagnosis and treatment of bone metastases for breast cancer patients is of great importance. Serum tumor and bone markers examinations have the priority of repeatable, non-invasive, and low in cost for patients. Thus, we analyzed the clinical characteristics of patients with bone metastases in breast cancer and clarify the risk factors for bone metastases.

Based on a large population analysis, the incidence of bone metastases from breast cancer at the primary diagnosis in our study was 15.33%, which was a little higher than that reported in Liede *et al*.’s study (13.2%)^7^. In addition, we found most of the patients with breast cancer were at the age of 40–49 years and were premenopausal status. These findings indicated that younger women with menstruation had a higher risk for developing breast cancer than those old patients with menopause. Purushotham *et al*.^8^ also reported that patients with breast cancer had a significantly decrease in the risk of developing distant metastasis with increasing age.

For the distribution of bone metastases, most of them were located in spine, especially in thoracic spine, followed by ribs, pelvis and sternum. The metastatic distribution in our study was in line with previous studies^9^. The reason for these is that breast carcinoma cells can easy migrate to neighboring ribs and spinal vertebrae via Batson venous plexus, and spread to the sternum via the parasternal lymph nodes. For the Batson venous plexus, it has no venous flap and is easy countercurrent^10^. In the present study, 46 patients were admitted to the hospital with bone metastases as the main symptoms, which were finally found to be breast cancer as the primary tumor. It suggested that breast cancer should be suspected if a female patient was identified with thoracic spine, ribs and sternum metastases.

In the current study, patients with breast cancer had a high incidence of axillary lymph node metastases, especially in bone metastatic patients. And the more axillary lymph nodes were involved, the higher probability of bone metastases was (Table 1). Additional analysis showed that axillary lymph node metastasis was one of the risk factors for bone metastases in breast cancer patients. Colleon *et al*.^11^ also found that patients who had four or more involved axillary nodes at the time of diagnosis had the highest incidences of bone metastases in breast cancer. It seems that the number of axillary node metastases was an important predictor for bone metastases in breast cancer.

In addition, we also identified the serum levels of CA125, CA153 and ALP were the risk factors for detecting bone metastases in patients with breast cancer. CA125 is well known as a tumor marker for ovarian cancer, but rarely evaluated in the early detection of bone metastases in breast cancer^12^. Berruti *et al*.^13^ found that the survival of patients with CA125 < 35 u/ml was significantly higher than those with CA125 > 35 u/ml in breast cancer. Baskic *et al*.^14^ reported that the positive rate of CA125 detection in breast cancer was 5%, but in distant metastases was 45%. In our study, the concentration of CA125 in patient with bone metastases was significantly higher than those without bone metastases. And the sensitivity and specificity for predicting bone metastases by CA125 were 61.66% and 83.36%, which were similar to Baskic *et al*.’s study.

CA153 is shown to be highly sensitive for distant metastases, especially in the bone and liver^15^. At present, it is recognized as a breast cancer tumor marker, which can be used as an effective indicator for the prognosis of bone metastasis in breast cancer^16^. The results of our study indicated that abnormal high CA153 level significantly increased the risk of bone metastases in breast cancer. And the cut-off value of it was 23.25 U/ml in the present study, which was reported to be ranged from 22 to 60 u/ml in previous studies^17, 18^. In line with Wojtacki *et al*. study^19^, the sensitivity of CA153 for diagnosing bone metastases in our study was 76.62%, and the specificity of it was 86.97%.

ALP is a bone formation marker, which is the most used marker for detecting increased bone formation in metastatic breast and prostate cancer^20^. Ritzke *et al*.^21^ found ALP in combination with CA153 led to the greatest sensitivity and positive predictive value for bone metastases in breast cancer. In the present study, the concentration of ALP was also identified as a risk factor for bone metastases in breast cancer patients, with a cut-off value > 100.5 u/l.

Previous study has reported that lack of hemoglobin-containing red blood cells is a common complication affecting more than 50% of cancer patients^22^. Kawai *et al*.^23^ found a component in bone marrow specifically related to the metastasis of prostate cancer to the bone and identified it as hemoglobin. Henke *et al*.^24^ reported that hemoglobin concentration was prognostic importance for patients with early breast cancer. In our study, the level of hemoglobin was significantly low in breast cancer patients with bone metastases compared to those without bone metastases. And it was identified as one of the risk factor for bone metastases in breast cancer. The reason for this may be bone metastases would lead to bone marrow dysfunction, which resulted in decrease of the hematopoietic function. However, the predictive accuracy for bone metastases by hemoglobin was low (AUC = 0.316).

Besides evaluating the single risk factor for predicting bone metastases in breast cancer, we also assessed the predictive accuracy of combining risk factors in this study. Based on the analysis, combined axially lymph node metastases, CA153, ALP with hemoglobin were found to be the most accurate one for predicting bone metastases in patients with breast cancer.

The limitations were also existed in the present study. First, this study was carried out just relying on a single-institutional database, although eligibility criteria were formulated to minimize the selective bias. Second, it was a retrospective study and some of the clinical data were missed, which may affect the analysis results. Third, we just collected the data of patients with breast cancer at the time of diagnosis. Some data, such as patients’ survival rate and follow up were not included in this study. Thus, a prospective, multi-center study is helpful to verify the results of our study.

In summary, we carried out a retrospective study to investigate the clinical characteristics of patients with breast cancer and identify the risk factors for bone metastases based on a large population analysis. The incidence of bone metastases in breast cancer was 15.3%, and the most frequent sites of bone metastases were spine, ribs, pelvis and sternum. The axillary lymph node metastases and the serum concentrations of CA125 > 21.99 u/ml, CA153 > 25.42 u/ml, ALP > 100.5 u/l and hemoglobin <49 g/l were identified to be the risk factors for bone metastases in patients with breast cancer. Combined axially lymph node metastases, CA153, ALP with hemoglobin were more closed related to bone metastases. However, due to the retrospective methodology and insufficient data, a prospective, multi-center study is necessary to verify the results of this study.
